# Impact of Rheumatoid Arthritis on Alopecia: A Nationwide Population-Based Cohort Study in Taiwan

**DOI:** 10.3389/fmed.2020.00150

**Published:** 2020-04-28

**Authors:** Yi-Jung Chang, Yung-Heng Lee, Pui-Ying Leong, Yu-Hsun Wang, James Cheng-Chung Wei

**Affiliations:** ^1^Department of Pediatrics, Chang Gung Memorial's Hospital, Taoyuan, Taiwan; ^2^Chang Gung University College of Medicine, Taoyuan, Taiwan; ^3^Department of Health Services Administration, China Medical University, Taichung, Taiwan; ^4^Department of Public Health, China Medical University, Taichung, Taiwan; ^5^Department of Orthopedics, Cishan Hospital, Ministry of Health and Welfare, Kaohsiung, Taiwan; ^6^Department of Center for General Education, National United University, Miaoli, Taiwan; ^7^Department of Rheumatology, BenQ Medical Center, The Affiliated BenQ Hospital of Nanjing Medical University, Taichung, Taiwan; ^8^Department of Allergy, Immunology and Rheumatology, Chung Shan Medical University Hospital, Taichung, Taiwan; ^9^Institute of Medicine, Chung Shan Medical University, Taichung, Taiwan; ^10^Department of Medical Research, Chung Shan Medical University Hospital, Taichung, Taiwan; ^11^Graduate Institute of Integrated Medicine, China Medical University, Taichung, Taiwan

**Keywords:** alopecia, rheumatoid arthritis, cohort study, corticosteroids, disease-modifying antirheumatic drugs

## Abstract

**Objectives:** Studies on the relationship between rheumatoid arthritis (RA) and alopecia areata (AA) are limited. This study investigated the effect of RA on alopecia areata risk in a nationwide cohort study.

**Methods:** We analyzed 2000–2012 data from the Longitudinal Health Insurance Database in Taiwan. The follow-up period was extended up to the end of 2013. We defined RA as a diagnosis using International Classification of Diseases, Ninth Revision, Clinical Modification (ICD-9-CM) code 714.0 during at least three outpatient visits or one admission and the use of disease-modifying antirheumatic drugs (DMARDs) for >30 days. The enrollees with AA were identified using the ICD-9-CM code 704.01. We enrolled a comparison cohort comprising participants randomly matched by age and sex, with the same index date as that of the study cohort. Furthermore, we investigated alopecia risk by using Cox proportional-hazards regression models after propensity score matching for sex, age, comorbidities, and medication use.

**Results:** In total, 2,905 patients with RA (74% women, mean age: 51.9 years) and 2,905 controls were followed for 22,276 and 25,732 person-years, respectively. Alopecia risk was 2.64-fold (95% confidence interval = 1.47–4.76) higher in patients with RA than in patients without RA after age, sex, comorbidities, and medication use were adjusted for. In addition, patients with thyroid disease presented considerable alopecia risk. Patients with RA in the younger age group (20–40 years) had the highest alopecia risk.

**Conclusions:** Alopecia risk is significantly higher in patients with RA than in those without RA, particularly in the younger age group (20–40 years). RA assessment should be considered when examining patients with alopecia, especially young adults.

## Introduction

Alopecia occurs worldwide, with a lifetime incidence of ~2% in the general population (1). It is usually characterized by unpredictable, recurring, chronic, and nonscarring loss of hair on the scalp or other areas (2). Alopecia is not a cosmetic problem specifically because it is associated with several comorbidities that may affect the patients' overall health and cause considerable morbidity (3–5). According to epidemiologic studies, autoimmune disorders, including rheumatoid arthritis (RA), play a role in the comorbid condition associated with alopecia (6).

RA affects 0.5–1% of the general population (7). It is a progressive disease involving a chronic and abnormal inflammatory reaction of the immune system against body tissues (systemic), primarily attacking joints and possibly hair follicles, leading to hair loss (8). Alopecia may present early in patients with RA, even before the more classical form of RA is identified. Timely diagnosis and appropriate treatment by a multidisciplinary team of clinicians are crucial for preventing further manifestations and yielding beneficial long-term outcomes in patients.

However, epidemiological studies on the relationship between RA and alopecia are limited, with many such studies with the limitation of a small sample size. Moreover, no clear conclusions can be drawn from these studies. To fill this knowledge gap, we conducted a nationwide population-based cohort study to elucidate alopecia risk in patients with RA.

## Methods

### Data Source

The present nationwide population-based study was based on data retrieved from the National Health Insurance (NHI) Research Database (NHIRD). The NHI program, established in 1995, covers >98% of the Taiwanese population. NHIRD contains anonymized data linked from the NHI for epidemiological study, including patient demographic details; health care service data; medication dispensation data from hospitals, general practices, and community pharmacies; diagnostic codes of International Classification of Diseases, Ninth Revision, Clinical Modification (ICD-9-CM), and drug codes. NHIRD has been extensively used in several published epidemiological research studies. The Longitudinal Health Insurance Database is a data subset comprising 1 million randomly sampled data from NHIRD.

### Patients

Patients with RA were identified from NHIRD using the ICD-9-CM code 714.0. These codes have been adopted in epidemiological studies that used NHIRD data (9, 10). We first selected patients who had received the first diagnosis of RA between January 1, 2000, and December 31, 2012. RA is typically treated with two types of anti-inflammatory agents, nonsteroidal anti-inflammatory drugs (NSAIDs) in combination with glucocorticoids and disease-modifying antirheumatic drugs (DMARDs) (11). In Taiwan, several DMARDs are frequently used for treating patients with RA, including hydroxychloroquine, methotrexate (MTX), and sulfasalazine. The NHI Administration in Taiwan recommends that rheumatologists should prescribe biological DMARDs for patients with RA in whom treatment with conventional DMARDs for at least 6 months is not effective (12). To improve the diagnostic validity of the analysis, this study only included patients with RA under DMARDs for ≥30 days and those aged ≥20 years. The index date was the date of the patient's first RA diagnosis. To define the appropriate cohort of RA patients, patients with comorbidities known to induce alopecia comorbidities such as systemic lupus erythematosus (ICD-9-CM code 710.0), cancer (ICD-9-CM codes 140–208), atopic dermatitis (ICD-9-CM code 691), and psoriasis (ICD-9-CM code 696) were excluded (13, 14). The enrollees with alopecia areata (AA) were identified by a diagnosis by dermatologists by using the ICD-9-CM code 704.01. We also excluded patients who received a diagnosis of alopecia areata (ICD-9-CM code 704.0) before the index date.

### Matching and Covariates

**Figure 1:**
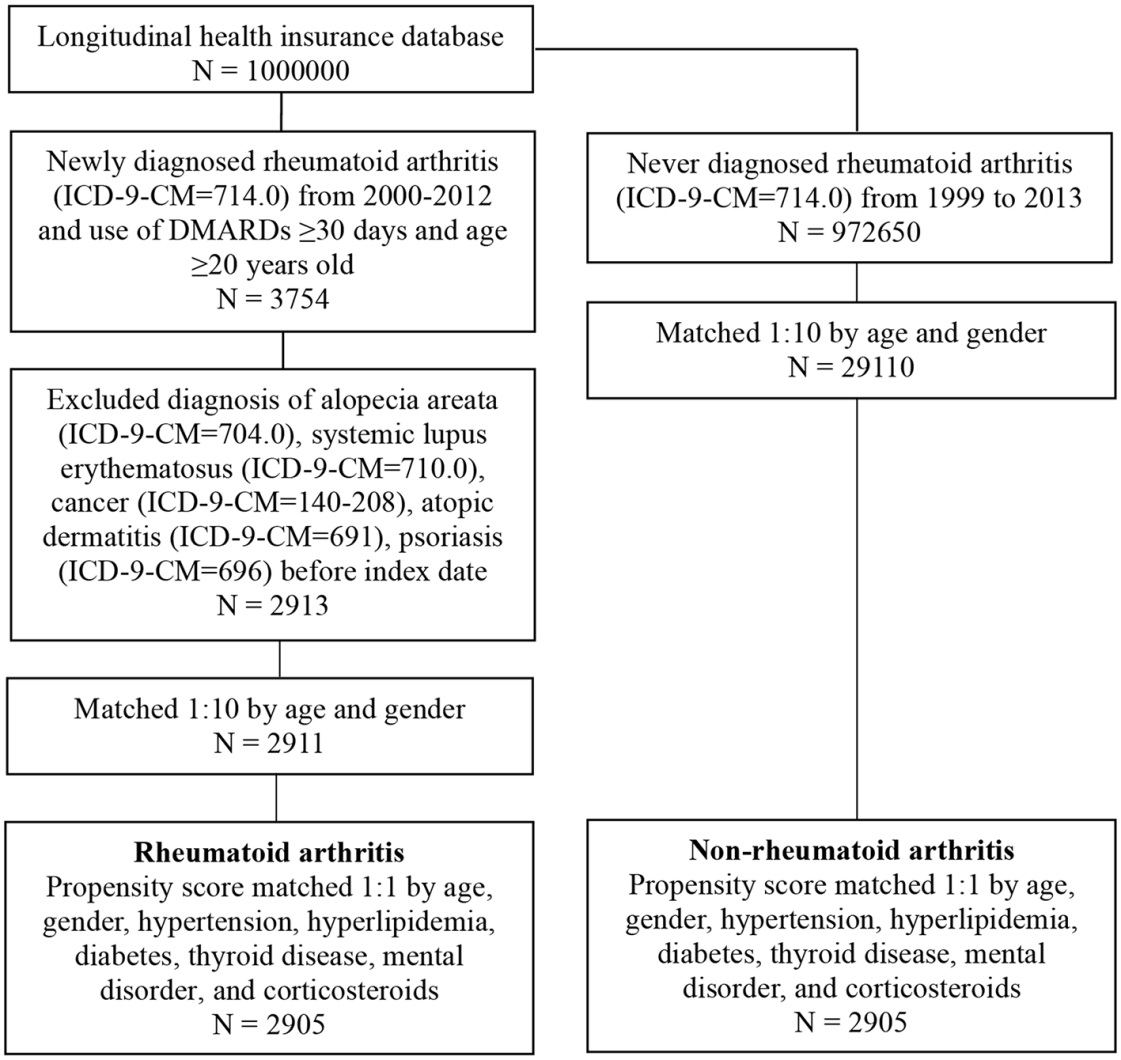
Study flowchart.

We conducted frequency matching to select individuals without RA as controls for comparison to increase the statistical power and control for potential confounding factors. Patients with RA and the comparison controls were 1:10 age and sex matched to obtain the same index date in both cohorts (Figure 1). We also estimated whether participants had any of these comorbidities: hypertension (ICD-9-CM codes 401–405), hyperlipidemia (ICD-9-CM codes 272.0–272.4), diabetes (ICD-9-CM code 250), and thyroid disorders (ICD-9-CM codes 240–246). The medical records of corticosteroid and DMARD prescription, including methotrexate and hydroxychloroquine, for ≥30 days were also retrieved from ambulatory and patient claims data for patients and controls. After 1:1 propensity score matching for age, sex, hypertension (ICD-9-CM codes 401–405), hyperlipidemia (ICD-9-CM codes 272.0–272.4), diabetes (ICD-9-CM code 250), thyroid disease (ICD-9-CM codes 240–246), mental disorder (ICD-9-CM code 290-319), and corticosteroid use in patients, we conducted the same matching on the comparison cohort to reduce the heterogeneity between the two groups. Information on comorbidity and medication use was determined at baseline and considered to be covariates in the multivariate analysis.

### Outcome Measurement

Outcome variables included AA (ICD-9-CM code 704.0) development during the follow-up period. To improve the diagnostic validity of the analysis, this study included only those patients who received a diagnosis of alopecia by a dermatologist. The patients and controls were followed until the occurrence of alopecia, December 31, 2013, or withdrawal from the national insurance system, whichever occurred first.

### Statistical Analysis

We used the χ^2^ test and Student's *t*-test to analyze the demographic data between the RA and non-RA groups and the Cox proportional-hazard regression models to calculate hazard ratios (HRs). The Kaplan–Meier curve was used to calculate the cumulative probability of AA in patients with RA. All data analyses were performed using SPSS (version 18.0; SPSS, Chicago, IL, USA), and *P* < 0.05 was considered statistically significant.

## Results

Before propensity score matching, 2,911 patients were included in the RA cohort and 29,100 individuals without RA were included in the control cohort. The RA and control cohorts did not significantly differ in terms of sex and age (Table 1). Patients with RA had a higher prevalence of pre-existing comorbidities such as hypertension, hyperlipidemia, chronic obstructive pulmonary disease, thyroid disease, and mental disorder (*P* < 0.05). Finally, after propensity score matching, 2,905 patients were included in the RA cohort and 2,905 participants with no RA diagnosis were included in the comparison cohort. The age in the propensity score-adjusted cohort was not statistically different between groups.

**Table 1:** Demographic characteristics of rheumatoid arthritis and non-rheumatoid arthritis.

	**Before propensity score matched**					**After propensity score matched**				
	**Rheumatoid arthritis (*****N*** **=** **2,911)**		**Non-rheumatoid arthritis (*****N*** **=** **29,110)**		***p*-value**	**Rheumatoid arthritis (*****N*** **=** **2,905)**		**Non-rheumatoid arthritis (*****N*** **=** **2,905)**		***p*-value**
	***n***	**%**	***n***	**%**		***n***	**%**	***n***	**%**	
Age					1					0.270
20–40	578	19.9	5,780	19.9		576	19.8	572	19.7	
40–65	1,797	61.7	17,970	61.7		1,794	61.8	1,750	60.2	
≥65	536	18.4	5,360	18.4		535	18.4	583	20.1	
Mean ± SD	51.9 ± 14.2		51.9 ± 14.2		1	51.9 ± 14.2		52.2 ± 14.4		0.327
Gender					1					0.472
Female	2,145	73.7	21,450	73.7		2,140	73.7	2,164	74.5	
Male	766	26.3	7,660	26.3		765	26.3	741	25.5	
Hypertension	612	21.0	5,256	18.1	<0.001	610	21.0	611	21.0	0.974
Hyperlipidemia	274	9.4	2,076	7.1	<0.001	271	9.3	271	9.3	1
Diabetes	228	7.8	2,378	8.2	0.526	226	7.8	221	7.6	0.806
COPD	130	4.5	725	2.5	<0.001	129	4.4	124	4.3	0.748
Thyroid disease	114	3.9	541	1.9	<0.001	108	3.7	112	3.9	0.783
Mental disorder	437	15.0	2,604	8.9	<0.001	433	14.9	424	14.6	0.739
Corticosteroids	2,246	77.2	6,009	20.6	<0.001	2,240	77.1	2,240	77.1	1
Methotrexate	1,175	40.4	38	0.1	<0.001	1,170	40.3	11	0.4	<0.001
Hydroxychloroquine	2,244	77.1	136	0.5	<0.001	2,239	77.1	37	1.3	<0.001

**Figure 2:**
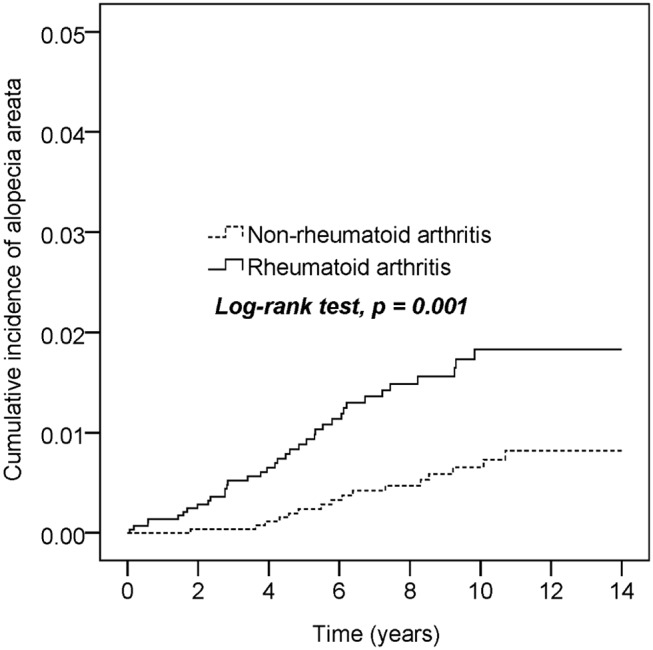
Cumulative incidence comparison of alopecia areata for patients with or without rheumatoid arthritis.

Table 2 presents the incidence density rate and adjusted HR (aHR) of alopecia in the study population. Patients with RA and controls were followed for 22,276 and 25,792 person-years, respectively. The overall alopecia incidence density rate was significantly higher in patients with RA than in controls (1.7 vs. 0.6 per 1,000 person-years). After adjustments for the covariates, alopecia risk was higher in patients with RA than in controls (aHR = 2.64, 95% confidence interval [CI] = 1.47–4.76). Other risk factors for AA included young age (20–40 years) and thyroid disease. Those aged ≥65 years had a lower AA risk than those aged 20–40 years (aHR = 0.15, 95% CI = 0.03–0.67). Patients with oral corticosteroids treatment had a lower alopecia risk (aHR = 0.57, 95% CI = 0.32–1.00) than those without. Table 3 presents the sex- and age-stratified effect of RA on alopecia. Female patients with RA probably had a higher AA risk than female patients without RA. Overall, patients with RA aged 20–40 or ≥40 years exhibited increased alopecia risk. Figure 2 presents the Kaplan–Meier curve, which indicates a higher cumulative incidence of alopecia in patients with RA than in controls (*P* = 0.001, log-rank test).

**Table 2:** Comparison of incidence and HR of alopecia between patients with and without rheumatoid arthritis by Cox proportional hazard model.

	**No. of alopecia areata**	**Observed person-years**	**Incidence Density (Per 1,000 person-years)**	**Crude HR**	**95% CI**	**Adjusted HR^†^**	**95% CI**
**Rheumatoid arthritis**							
No	16	25,792	0.6	1		1	
Yes	37	22,276	1.7	2.67	1.49–4.81	2.64	1.47–4.76
**Age**							
20–40	27	10,426	2.6	1		1	
40–65	24	29,797	0.8	0.31	0.18–0.53	0.35	0.20–0.63
≥65	2	7,846	0.3	0.10	0.02–0.41	0.15	0.03–0.67
**Gender**							
Female	38	36,190	1.1	1		1	
Male	15	11,879	1.3	1.21	0.67–2.21	1.11	0.61–2.05
Hypertension	4	8,921	0.4	0.35	0.13–0.98	0.77	0.26–2.28
Hyperlipidemia	2	3,885	0.5	0.44	0.11–1.82	0.81	0.19–3.47
Diabetes	1	3,200	0.3	0.27	0.04–1.93	0.50	0.07–3.81
Thyroid disease	5	1,764	2.8	2.75	1.09–6.91	3.53	1.38–9.05
Mental disorder	3	6,476	0.5	0.38	0.12–1.22	0.50	0.15–1.63
Corticosteroids	34	38,626	0.9	0.44	0.25–0.77	0.57	0.32–1.00

† *Adjusted for age, gender, hypertension, hyperlipidemia, diabetes, thyroid disease, mental disorder, and corticosteroids*.

**Table 3:** Subgroup analysis of Cox proportional hazard model stratified by sex and age.

	**Rheumatoid arthritis**		**Non-rheumatoid arthritis**			
	***N***	**No. of alopecia areata**	***N***	**No. of alopecia areata**	**HR**	**95% CI**
**Age**						
20–40	576	18	572	9	2.28	1.03–5.08
≥40	2,329	19	2,333	7	3.13	1.31–7.45
					*p* for interaction = 0.144	
**Gender**						
Female	2,140	29	2,164	9	3.75	1.77–7.92
Male	765	8	741	7	1.29	0.47–3.57
					*p* for interaction = 0.164	

## Discussion

This is the first nationwide population-based study to assess the association between RA and alopecia risk. This study demonstrated important findings. First, we found a 2.6-fold increased alopecia risk in patients with RA compared with the population without RA, especially in younger age groups (20–40 years). Second, both RA and thyroid disease occurred significantly more frequently in patients with alopecia. Furthermore, we noted a probably negatively associated determinants of alopecia in oral corticosteroids for patients with RA.

Although patients with RA in this study had a higher prevalence of comorbidities and coexistent conditions associated with alopecia development than the comparison cohort, RA remains an independent risk factor for alopecia after adjustments for covariates. Several biological lines of evidence support this statistical outcome. Petukhova et al. (15) demonstrated that 139 single-nucleotide polymorphisms are associated with alopecia and mostly related to inflammation and immunomodulation. Genome-wide association studies have revealed that autoimmune diseases (e.g., alopecia, RA, and type 1 diabetes mellitus) involve similar pathological changes within the Janus kinase and signal transducer and activator of transcription signaling (16). Mouse model studies have also indicated the role of an identical pathogen involved in this signaling in alopecia (17, 18).

Autoimmune diseases, including RA, could be a crucial risk factor for alopecia. Moreover, Cox proportional-hazards regression analysis results in the present study demonstrated a significant increase in alopecia risk in not only patients with RA but also patients with thyroid disease. Moreover, alopecia incidence rate increased in all age groups in patients with RA compared with those without. This finding is consistent with previous studies that showed that thyroid disease is the most common autoimmune comorbidity in patients with alopecia (19, 20). A retrospective study on children with alopecia screened for thyroid function revealed that 20% of the analyzed patients had abnormal thyroid test results (21). Several studies have found the presence of thyroid autoantibodies in 15–20% of patients with alopecia (2, 22, 23). Our study clarified that thyroid disease and RA were most significantly associated with alopecia. Previous studies have concluded that thyroid function screening should be considered, particularly in alopecia patients with a history of Down syndrome or atopy or a family history of thyroid disease (2). Furthermore, our study results can help clinicians identify and understand comorbidities, which can enhance their ability to recommend screening and preventative measures.

In the general population, alopecia can develop at any age; however, in 82.6–88% of the cases, it develops by the age of 20 years (24, 25). This study demonstrated that the young age was associated with a lower alopecia risk. Genetic (inherited) factors may play a role in the interaction between alopecia and age (26). Early onset of AA seems to indicate the underlying genetic background. Chu et al. (13) recently found that AA onset age predicts the associated autoimmune diseases. Almost 40% of individuals aged <30 years with AA have at least one family member who has been diagnosed with the same disorder (27).

A notable finding of the current study is that oral glucocorticoid use was probably associated with a reduced AA risk. Systemic glucocorticoids are occasionally prescribed as a temporary measure to slow hair loss in patients with rapidly progressing extensive hair loss (28, 29). By contrast, some DMARDs used in RA management may present adverse effects such as hair loss (30, 31). Alopecia was reported as an adverse effect of treatment with methotrexate. In the present study, the clinical suitability of glucocorticoids or DMARDs for disease activity assessment remains uncertain.

We found significant associations between RA and alopecia, indicating the crucial role of RA in alopecia. Although our study also demonstrated other determinants of alopecia included thyroid disease, young age (positively associated), and glucocorticoid therapy (negatively associated), there are some potential limitations: First, because of the large variation in clinical manifestations and diagnostic definitions, RA diagnosis may be disregarded by clinicians. However, the accuracy of RA and AA diagnoses in NHIRD has been validated previously (9, 10, 13). Second, NHIRD does not include data on covariates, such as family history, personal lifestyle, social adversity, laboratory data, environmental factors, and disease activity. The RA disease activity may have an impact in alopecia. The lack of data on disease activity and glucocorticoid/DMARD doses is also a limitation.

In conclusion, RA is associated with a higher incidental alopecia risk. Alopecia risk must be carefully evaluated in patients with RA. Clinically, understanding these findings is of high relevance for damage prevention. Further prospective studies should investigate the cost-effectiveness of screening for RA in patients with alopecia.

## Data Availability Statement

The datasets generated for this study are available on request to the corresponding author.

## Ethics Statement

This study was approved by the Chung Shan Medical University Hospital Ethics Committee.

## Author Contributions

JW designed the research. Y-JC and Y-HL were responsible for drafting the manuscript and collected the data. Y-HW and P-YL analyzed the data. Y-JC and JW reviewed and revised the manuscript. Y-HL, P-YL, and Y-HW reviewed the manuscript. All authors approved this version to be published.

## Conflict of Interest

The authors declare that the research was conducted in the absence of any commercial or financial relationships that could be construed as a potential conflict of interest.
